# Using AI to enhance healthcare resource management and allocation: A focus on the autism community in Alabama

**DOI:** 10.1371/journal.pone.0342700

**Published:** 2026-03-16

**Authors:** Armin Ahmadi, Jerome Baudry, Nathan Tenhundfeld, Kelly Goff, Daniel Adamek

**Affiliations:** 1 Department of Biological Sciences, The University of Alabama in Huntsville, Huntsville, Alabama, United States of America; 2 Department of Psychology, The University of Alabama in Huntsville, Huntsville, Alabama, United States of America; 3 Autism Services, Alabama Department of Mental Health, Montgomery, Alabama, United States of America; 4 Little Orange Fish, Huntsville, Alabama, United States of America; Federal University of Paraiba, BRAZIL

## Abstract

This study investigates the potential of artificial intelligence, particularly Natural Language Processing and large-scale language models, to improve resource management and service access for individuals with autism in Alabama. The research aims to explore and evaluate the potential of AI-driven tools to address challenges in navigating complex datasets and supporting social work practices. We designed and tested AI systems, including general language models, domain-specific chatbots powered by advanced language models, and a Retrieval-Augmented Generation framework. A standardized set of queries was used to simulate real-world scenarios encountered by social workers engaged in autism service coordination. System performance was evaluated based on precision, recall, and response accuracy. Results demonstrated that the Retrieval-Augmented Generation (RAG) framework achieved superior performance compared to traditional NLP methods and general-purpose language models, attaining approximately 90–96% precision and recall across evaluated query types. RAG outperformed the domain-specific GPT-4 chatbot by approximately 5–12 percentage points in F1 score, with the largest gains observed for queries requiring geographic specificity, multiple constraints, or complex contextual understanding. The integration of domain-specific retrieval significantly enhanced the accuracy, contextual relevance, and usability of generated responses. The findings highlight the transformative potential of AI-driven tools in improving social work efficiency and enhancing healthcare equity. By streamlining care coordination and delivering accurate, contextually relevant information, these systems offer scalable solutions to improve access to autism-related services. Future research should focus on addressing data quality, minimizing biases, and ensuring ethical deployment to build trust and support widespread adoption of these tools.

## Introduction

Navigating fragmented healthcare systems presents significant challenges for patients, caregivers, and associated services, particularly for those managing chronic or complex conditions like autism. These inefficiencies often result in unmet medical needs and reduced patient satisfaction [1]. Integrated care, which emphasizes coordination and communication among healthcare providers, has consistently demonstrated improvements in patient outcomes and satisfaction by streamlining services and reducing redundancies [2,3]. Holistic care platforms further enhance healthcare efficiency and sustainability, though implementing such systems is not without obstacles [4]. These challenges are particularly pronounced in the context of Autism Spectrum Disorder (ASD), where service navigation requires coordination across healthcare, education, social services, and community-based organizations. In Alabama, families and social workers supporting individuals with autism must navigate a fragmented landscape of providers with heterogeneous eligibility requirements, insurance acceptance policies, geographic availability, and age-specific service constraints. This complexity often leads to delays, service gaps, and increased caregiver burden, especially in rural and underserved regions where provider availability is limited. As a result, identifying appropriate, local, and accessible autism-related services remains a persistent and practical challenge for both families and social workers.

Human social workers play a pivotal role in care coordination but often face overwhelming workloads due to high caseloads and undefined roles, which can limit their effectiveness [5]. Furthermore, only 30% of healthcare systems report robust collaborations with community-based service providers, exacerbating inefficiencies in care delivery [6]. Issues such as human error and limited information processing further hinder case management, making it difficult to monitor outcomes and ensure accountability [7]. While addressing social needs within clinical care demonstrates a strong return on investment, fragmented systems continue to drive high financial and human costs [8].

Artificial Intelligence (AI) emerges as a particularly promising tool for addressing these challenges in autism service navigation, where large volumes of heterogeneous, unstructured information must be queried efficiently and accurately. By processing large datasets, identifying patterns, and providing evidence-based insights in real time, AI improves resource management and care coordination [9,10]. Automating administrative tasks like scheduling and record-keeping reduces inefficiencies and human error, with McKinsey estimating up to a 30% reduction in healthcare costs through AI-driven operational improvements [11]. Beyond administrative efficiencies, AI also enables personalized care through data analysis, delivering tailored interventions that enhance treatment outcomes [12]. For instance, during the COVID-19 crisis, AI-powered tools effectively guided patients to appropriate care, demonstrating the potential for streamlined coordination and improved outcomes [13]. Global applications further illustrate how AI can optimize resource allocation, reduce delays, and minimize redundant treatments [14,15]. Social workers, in particular, benefit from AI’s ability to track interventions, forecast challenges, and allow for a focus on higher-level care needs [16].

Autism Spectrum Disorder (ASD) exemplifies a domain where these systemic challenges are particularly acute, making it a compelling use case for evaluating AI-driven approaches to resource navigation and care coordination. ASD management requires a coordinated, multidisciplinary approach due to its complexity and the necessity for collaboration among diverse stakeholders [17]. Effective care transitions, particularly from childhood to adult services, remain critical but are often hampered by personnel shortages and systemic inefficiencies [18]. Socioeconomic and cultural factors further compound these challenges, necessitating adaptive and equitable interventions [19]. The prevalence of ASD has significantly increased, with the CDC reporting that one in 36 children in the U.S. is diagnosed with ASD, a trend mirrored in Alabama with a 22% rise in prevalence since 2021 [20,21]. Navigating autism-related services presents additional challenges due to complex eligibility requirements, insurance constraints, and a fragmented landscape of healthcare, education, and community resources. Families and social workers often face difficulties coordinating services across these sectors, which leads to delays or gaps in accessing vital services. These challenges are especially pronounced in rural and underserved communities, where resource scarcity intersects with geographic and economic barriers [22,23]. Families of autistic individuals frequently report high caregiver burden due to the constant navigation of disconnected systems, including healthcare providers, educational institutions, therapeutic services, and legal advocates [24]. These barriers result in inequities in care and missed opportunities for timely intervention.

Social workers play a critical role in supporting individuals with ASD and their families by coordinating care across healthcare, education, insurance, and community-based service systems. In collaboration with the Alabama Department of Mental Health, this study focused on social workers as the primary users because they are directly responsible for navigating fragmented service landscapes, identifying appropriate providers, and verifying eligibility constraints on behalf of clients. These responsibilities make social workers a natural and practically accessible user group for evaluating AI-driven resource navigation tools. Accordingly, AI-assisted systems that streamline data access and support multi-criteria service matching have the potential to directly enhance social work efficiency and care coordination [25]. By integrating diverse datasets, AI offers a holistic view of each client’s needs, leading to tailored care plans and improved collaboration among service providers. Studies show a generally positive reception toward AI tools in healthcare, with about 67% of internet users expressing acceptance of AI-led services [26]. However, successful integration of AI requires targeted training to navigate challenges in prediction, adaptivity, and decision-making [27].

By focusing specifically on autism service navigation in Alabama, this study illustrates how AI-driven tools can address real-world challenges faced by social workers coordinating care across fragmented service ecosystems. AI technologies empower social workers by streamlining care coordination, enabling more personalized and effective support, and addressing critical gaps in service delivery. Here, we focused specifically on social workers as the primary end users because the tool was developed as a proof-of-concept social worker assistant. This work leverages such use cases, workflows, and data sources, and provide a basis for the extension of this work to other stakeholders such as caregivers, physicians, and educators. This research provides a scalable model for leveraging AI to improve care for underserved populations, highlighting its potential to enhance resource allocation, reduce systemic inefficiencies, and deliver better outcomes across complex healthcare systems. The system was designed and evaluated specifically as a proof-of-concept social worker assistant, with workflows, query design, and evaluation criteria optimized for social work use cases in autism care coordination. While improved navigation may indirectly benefit families, caregivers, physicians, and educators, these groups are considered secondary beneficiaries rather than primary end users. The present study therefore centers on social workers as the intended users, while providing a foundation for future extensions to additional stakeholder groups

## Background and technical foundations: motivation for system design

### Leveraging AI in healthcare resource management

Artificial Intelligence (AI), particularly Natural Language Processing (NLP) and Large Language Models (LLMs), has emerged as a transformative force in healthcare resource management. These technologies efficiently process large volumes of unstructured data, such as clinical notes and patient records, which are often underutilized in traditional systems [28]. For example, NLP applications in radiology have demonstrated 95–96% consistency in decision-making [29], illustrating the potential of structured language processing in healthcare while also highlighting the domain-specific nature of such performance gains. NLP and LLMs extract critical insights from unstructured data, enhancing treatment planning, clinical trial recruitment, and patient engagement [30]. LLMs, particularly when integrated into Clinical Decision Support Systems (CDSS), further revolutionize care delivery by leveraging IoT devices for real-time insights [31].

### Traditional NLP in healthcare: strengths and limitations

Traditional NLP systems rely on rule-based algorithms and machine learning models to perform tasks like medical coding and information extraction, often achieving high accuracy in specific applications. For instance, an XGBoost classifier achieved an F1 score of 0.8881 in diagnosing psychosis from psychiatric notes, and other models have reached 99% accuracy in classifying PHI-related data [32,33]. However, these systems struggle with scalability, contextual understanding, and adaptability to new or complex medical terminology. Scalability challenges, particularly in cloud-based environments, can increase latency by up to 50% [34]. Additionally, traditional models experience performance declines of 15–20% when faced with new ICD codes without retraining [35]. Manual feature engineering is another limitation, requiring intensive human effort that can be reduced by up to 70% with deep learning models [36]. These systems also fail to interpret nuanced queries effectively, underperforming by 20–30% compared to transformer-based models like BERT in tasks involving complex language patterns [37]. These limitations, particularly reduced performance on nuanced, multi-criteria queries, are directly relevant to autism service navigation, where users often express needs using heterogeneous terminology, incomplete phrasing, and multiple constraints. This motivated our inclusion of a traditional NLP-based chatbot as a realistic baseline rather than a domain-optimized solution

### Advancements with LLMs: benefits and challenges

LLMs, including GPT models, represent significant advancements in language processing due to their transformer-based architectures and ability to handle complex, context-rich queries with minimal retraining [38]. BERT-based models, for instance, improved patient record summarization accuracy by up to 20−30% over traditional approaches [39]. These models excel in few-shot or zero-shot learning scenarios, reducing the need for extensive fine-tuning, with GPT-3 demonstrating a 30% improvement in task accuracy using natural-language prompts [40]. Furthermore, studies have shown that LLM-generated summaries are preferred over human-generated ones by up to 40% for their completeness and correctness [41].

Despite these advantages, LLMs require significant resources for fine-tuning and deployment. A fine-tuned GPT-3.5 model, for example, achieved an F1 score of 0.80 compared to 0.46 for an out-of-the-box version, but such training can cost millions of dollars [14,42]. Biases in training data and the “black box” nature of LLMs also pose challenges for trust and interpretability, with studies revealing significant biases in applications like physician use cases [43,44]. Additionally, prompt engineering plays a critical role in optimizing LLM performance, particularly in specialized domains like healthcare, where well-designed prompts can achieve nearly 90% precision and recall [45].

Importantly, the performance improvements reported in prior LLM studies primarily reflect gains in general language understanding rather than localized service retrieval. In the context of autism resource navigation, these characteristics suggest that while LLMs may better interpret complex user queries than traditional NLP systems, they may still struggle to provide accurate, region-specific, and actionable recommendations without access to curated local data. This distinction motivated our evaluation of both out-of-the-box LLMs and a domain-specific GPT-4 implementation.

### Retrieval-augmented generation (RAG): enhancing LLMs

Retrieval-Augmented Generation (RAG) combines the generative capabilities of LLMs with dynamic information retrieval, offering a mechanism to translate general language understanding into accurate, domain- and location-specific responses—an essential requirement for autism service navigation. By incorporating real-time domain-specific data, RAG systems address the limitations of static LLMs, such as knowledge gaps and biases. For instance, BiomedRAG achieved micro-F1 scores of 81.42 and 88.83 in healthcare tasks, surpassing traditional NLP systems [46]. This approach also enhances transparency in decision-making, as demonstrated in its success managing chronic Hepatitis C Virus infections and reducing biases in handling sensitive data [47,48].

RAG systems are highly scalable and efficient for time-sensitive healthcare scenarios. In one preoperative medicine case study, an LLM-RAG model processed 1,260 responses using 35 guidelines within 15−20 seconds, compared to the 10 minutes required by human experts. This model improved GPT-4’s accuracy from 80.1% to 91.4%, exceeding human performance [49]. By dynamically integrating retrieved documents into LLM outputs, RAG enhances open-domain question answering and supports better patient outcomes [50].

Together, these prior findings informed the comparative system design evaluated in this study. Specifically, we examined (i) a traditional NLP-based chatbot reflecting commonly deployed directory search tools, (ii) general-purpose and domain-specific LLM-based chatbots leveraging improved language understanding, and (iii) a RAG-based system designed to integrate local, curated autism service data with generative reasoning. This progression reflects a deliberate mapping from known limitations to proposed solutions rather than a comparison against external domain benchmarks.

## Methodology

This study develops an AI-driven model to improve resource management and support social workers in autism care in Alabama. By integrating diverse primary (healthcare providers), secondary (social services), and tertiary (community organizations) stakeholders, the model tries to address inefficiencies, enhances care coordination, and improves access to services. Key stakeholders were identified and categorized (S1 Table), and their roles and interactions mapped to reveal gaps in existing systems to feed the model with a broad understanding of healthcare systems and public services, incorporating typical roles and responsibilities from healthcare, social services, education, legal services, and other relevant areas.

### Data collection and integration

To construct a comprehensive database of autism-related service providers in Alabama, we employed a combination of manual data collection and automated web scraping during February 2024. Publicly accessible sources included provider directories, organizational websites, nonprofit listings, and state-maintained documents related to autism services. Automated scraping was used to extract structured information (e.g., provider name, location, service descriptions) where feasible, while manual review and curation were applied to validate entries and capture information from sources not amenable to automated extraction. This process identified 513 unique providers, and collaboration with the Alabama Department of Mental Health, which also informed the study’s focus on social work–driven service navigation, added an additional 1,102 service-related entities compiled from over 350 textual documents, including therapy providers, community organizations, schools, nonprofit advocacy groups, and public agencies relevant to autism care in Alabama. Service providers were included if they offered autism-related healthcare, therapeutic, educational, social, legal, or community-based services within Alabama and had a verifiable operational presence (e.g., physical address or documented service region). Entries lacking sufficient identifying information, duplicative listings, or services unrelated to autism care were excluded. In this context, “providers” refer to both individual practitioners and organizations, with each database entry representing a unique service unit rather than an individual client or case. This resulted in a final dataset of 1,615 entries. Duplicate records were removed using a two-step process: first, through exact matching of provider names and addresses, and second, through approximate (“fuzzy”) string matching to identify minor variations or typographical differences (e.g., “St.” vs. “Street”). This approach reduced redundancy while preserving distinct service units. The database supported both chatbot systems (Athena and Minerva) used in this study. In this context, “providers” refers to both individual practitioners and organizations offering autism-related services. Each provider’s entry represents a unique service unit with a verifiable address and operational role. Key details such as provider names, addresses, contact information, and service descriptions were digitized using Optical Character Recognition (OCR) software [51]. Outputs generated through OCR and GPT-4–assisted conversions were manually reviewed to validate critical fields, including provider identity, service offerings, and geographic location. Data originally in unstructured formats—such as PDFs and handwritten notes—were converted into structured CSV files using GPT-4 [52] to improve standardization and consistency. Manual validation ensured that digitization errors did not propagate into downstream analyses. Because the study was designed as a proof-of-concept evaluation, data collection emphasized breadth, verifiability, and practical relevance over exhaustive enumeration of all possible service providers.

### Service provider tagging

A tagging system was developed to facilitate querying and data retrieval. Each service provider was assigned binary tags (‘1’ for offered services, ‘0’ for non-offered) across 300 categories (example in Table 1), covering diverse needs of the autism community, such as insurance, healthcare services, therapeutic supports, and education [53]. The binary tagging system was selected to enable scalable and interpretable matching between user queries and provider offerings. “Offered services” were defined as those that providers publicly listed or were documented as delivering in source materials. When services were ambiguous or conditional (e.g., only offered to certain age groups), we opted to create more specific tags (e.g., “early intervention” or “adult services”) rather than apply weighted labels. Some proprietary tags (e.g., “Cigna,” “United Healthcare”) were included to reflect insurance acceptance, which is a critical filtering dimension for many families. These were treated as separate but parallel attributes to broader categories such as “financial support.” Expert social workers reviewed the database to validate the accuracy and relevance of the tags. This review included an independent pass by a domain expert from the Alabama Department of Mental Health. Agreement between internal team annotations and the external expert’s review was high, with only a small number of disputed tags (e.g., < 5% of providers requiring clarification). Discrepancies were resolved by consensus discussion, ensuring consistent application of the tagging schema. This process provides qualitative support for the reliability of the tagging approach, though formal statistical measures of inter-rater agreement (e.g., Cohen’s κ) were not calculated given the proof-of-concept scope of this work.

**Table 1:** Sample categorization of Service Providers for Autism Care.

Category	Tags
**Insurance & Financial**	Medicaid, AllKids, BCBS, Cigna, United, Tricare, Aetna, Medicare, Financial Supports
**Healthcare Services**	Pediatricians, Neurologists, Psychiatrists, Dentists, Nutritionists, Gastroenterologists, Pulmonologists, Endocrinologists, Dermatologists, Optometrists, Pediatric Neurologists
**Therapeutic Services**	Applied Behavior Analysis (ABA), Occupational Therapy, Physical Therapy, Speech Language Therapy, Behavioral Therapy, Art/Dance/Music Therapists, Aquatic Therapists, Equine Programs
**Mental & Emotional Support**	Psychologists & Counselors, Social Workers, Crisis Services, Support Groups, Substance Use Treatments, Counseling for Children, Teens, and Adults
**Education & Development**	Early Intervention Providers, Specialized Schools, Post-Secondary Education, Autism Evaluation, Autism Friendly Services, Educational Supports
**Legal & Advocacy**	Attorneys, Legal Services, Advocacy & Support, Autism Associations
**Community & Social Support**	Community Activities, After-school Programs, Camps, Social Skills Programs, Recreational & Community Activities, Housing & Community Living, Faith-Based Organizations
**Employment & Vocational**	Employment Supports, Vocational Rehabilitation, Job Training Programs
**Special Needs Services**	Assistive Technology, Genetic Testing, Safety/Protection, Transportation, Feeding Therapies/Interventions, Sleep Supports
**Crisis & Immediate Care**	Emergency Services, First Responder Resources, Inpatient Treatment Care Centers, Crisis Intervention Services
**Residential & Home Care**	Residential Programs, Home School, Day Programs, Housing Assistance

Table 1 presents a subset of representative service categories for illustrative purposes. The full tagging taxonomy comprises approximately 300 service-related categories spanning clinical, educational, financial, legal, and community-based domains. Certain proprietary insurance providers (e.g., Cigna, United) were treated as distinct tags rather than grouped under general financial support categories due to their practical importance for eligibility-based filtering in autism service navigation.

### Socioeconomic mapping of resources

To identify any possible disparities in autism-related service availability, a spatial analysis [54] was conducted by mapping service provider locations against socioeconomic factors obtained from the U.S. Census Bureau [55]. Key factors included population density, ethnic composition, median household income, and poverty rates. Custom Python scripts utilized the Census API for data retrieval, Pandas [56] for data organization, and Folium [57] for interactive mapping. Service provider addresses were geocoded using the Nominatim geocoder [58] via the GeoPy [59] Python library and presented in a map of resource accessibility and underserved areas.

### Development of the NLP-driven chatbot

**Fig 1:**
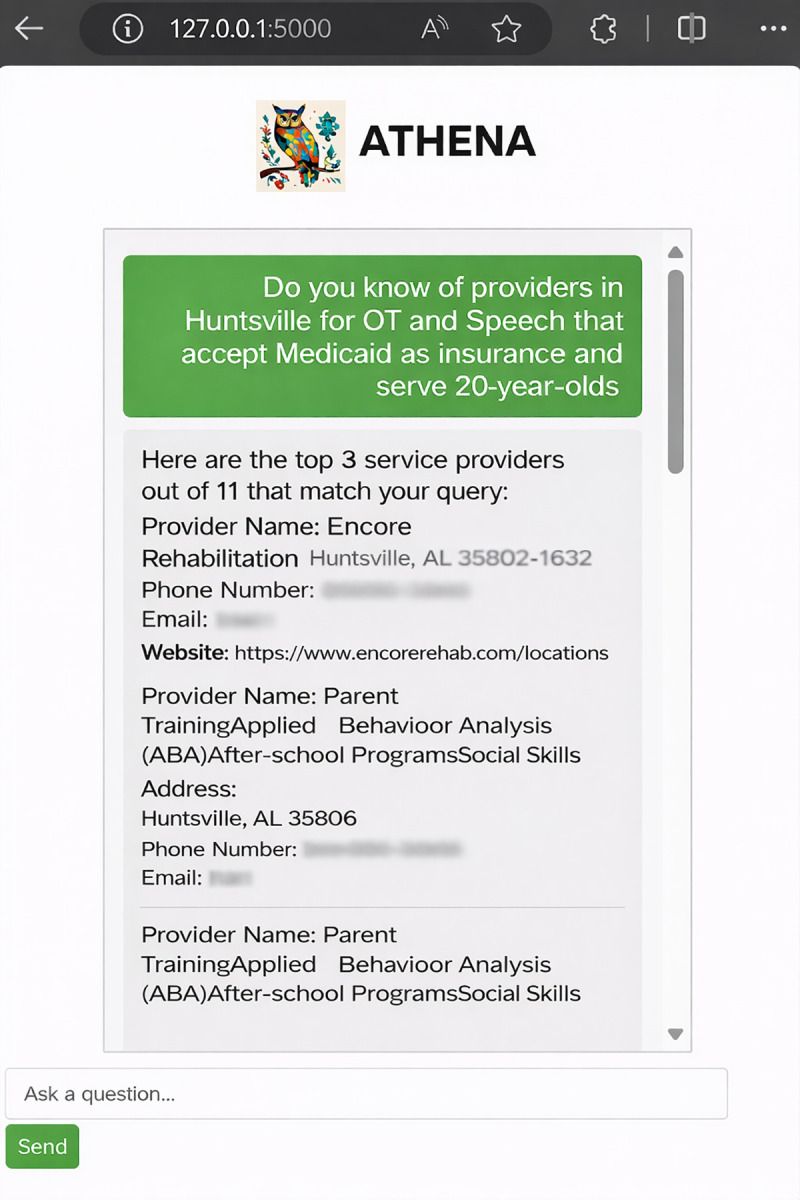
NLP-driven Chatbot response example, returning a filtered set of autism service providers based on user query criteria. Information shown was drawn from publicly available sources (e.g., provider websites) and manually blurred to remove identifying details. No personal health information or private individual data is presented..

An NLP-driven chatbot was developed to provide users with a tool to locate autism service providers based on specific needs and locations. This system was intentionally designed to reflect commonly deployed, rule-based and keyword-driven service directory tools rather than a state-of-the-art NLP model, and therefore serves as a pragmatic baseline for comparison. The service provider database, initially in CSV format, was serialized into JSON files to optimize data handling and interoperability with NLP tools [60]. In house Python script were used to create comprehensive provider profiles, encapsulating details such as name, address, services offered, and additional notes. The chatbot was built using Python 3.10 and the SpaCy v3.7 NLP library [61], utilizing the standard English NLP model (en_core_web_sm) for tasks such as tokenization, lemmatization, and named entity recognition (NER). The purpose of this baseline system was not to maximize NLP performance, but to model the behavior of realistic, widely used directory-style search tools that rely on predefined taxonomies, synonym normalization, and fuzzy string matching. Such systems remain prevalent in public health, social services, and nonprofit resource platforms, making them a relevant reference point for evaluating the added value of LLM- and RAG-based approaches. A custom-built service_synonyms dictionary was developed to normalize user queries across heterogeneous provider terminology. For example, terms such as “speech therapy,” “speech pathologist,” and “SLP” were grouped under a unified category (“Speech Language Therapists”). The dictionary was manually curated and informed by best practices in semantic alignment and concept disambiguation, but did not directly use external lexical resources such as WordNet or BabelNet. When exact matches were unavailable, fuzzy string matching with RapidFuzz Python library [62] with a score cutoff of 80% was employed. Geographic information was managed by extracting locations through SpaCy’s NER capabilities and geocoding user-provided locations with Python’s geopy library (v2.4.1), specifically leveraging Nominatim geocoder (OpenStreetMap) for location identification. The geodesic distance between users and identified service providers was calculated using geopy, prioritizing providers within a 50 km radius. The system incorporated error-handling mechanisms that ensured incomplete or failed geocoding queries did not eliminate providers from recommendations but rather marked distances as “Unknown” to maintain inclusiveness. The chatbot interacts through a Flask [63] hosted Linux server with Ubuntu 22.04 LTS (Fig 1), prompting users to enter queries in natural language. It provides structured responses that include provider information, services offered, and contact details, enhancing accessibility and usability.

### Advancements with LLM-powered chatbots

To explore the applicability of Large Language Models (LLMs) in enhancing autism care support, we first tested widely available generative AI models (out-of-the-box LLMs), including ChatGPT (OpenAI), Google Gemini, Microsoft Copilot, and Claude (Anthropic) which are recognized for their advanced natural language understanding, ability to handle complex and nuanced queries, and general versatility across a range of domains [64]. These models were assessed using a standardized set of queries designed to simulate scenarios encountered by social workers and families seeking autism-related services in Alabama. The queries aimed to evaluate their ability to handle complex, multi-criteria requests, such as identifying local service providers based on specific needs and insurance requirements.

**Fig 2:**
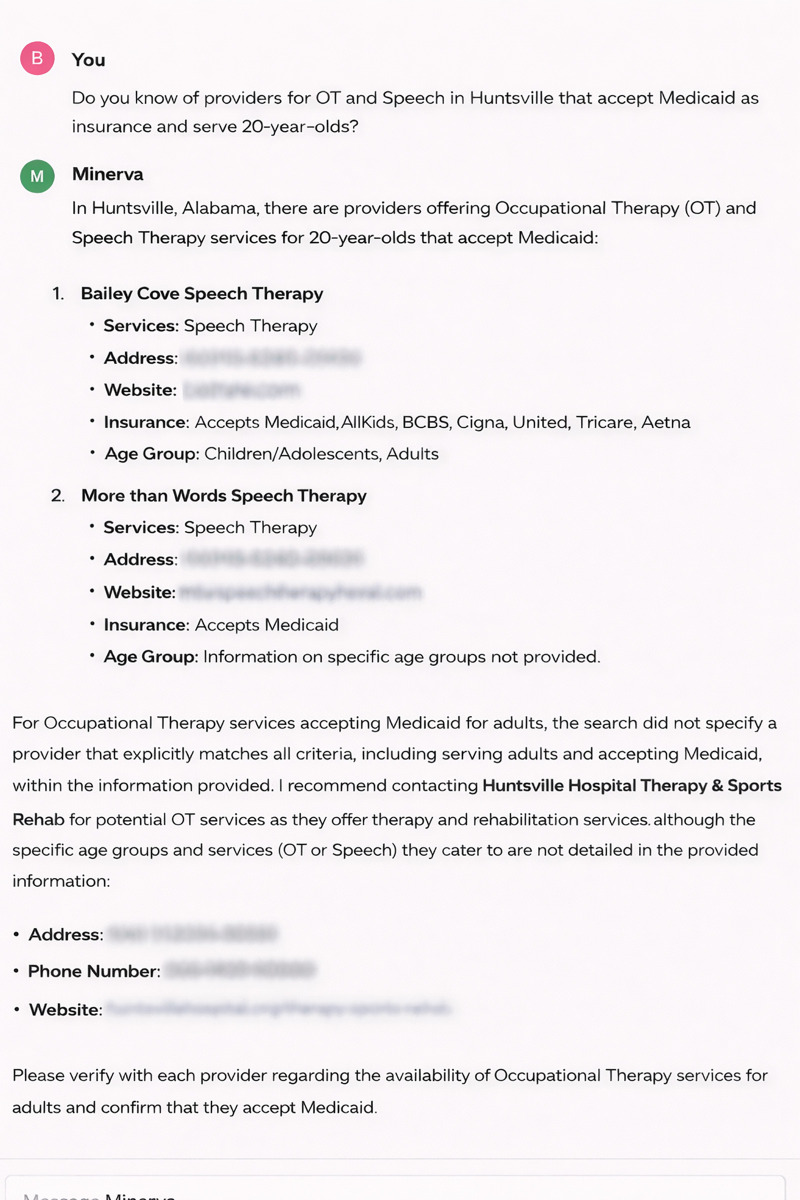
LLM driven Chatbot response example. Provider details are sourced from public records and have been blurred for privacy. No sensitive or user-specific information is shown.

Based on observed strengths and limitations, we developed a domain-specific chatbot named “Minerva,” built specifically upon OpenAI’s GPT-4 model (GPT-4 API, version gpt-4–1106-preview). The GPT-4 model was employed with carefully engineered prompt templates (Table 2), emphasizing concise, clear instructions explicitly linked to autism service criteria and geographic specificity within Alabama. Prompt engineering utilized simple few-shot prompting techniques, explicitly listing query-response examples to guide model behavior without further fine-tuning. The structured autism provider database described earlier, serialized as JSON, provided the domain-specific context for generating responses. Iterative refinement cycles integrated structured user feedback obtained through a binary (“Yes/No”) feedback loop built into the user interface to progressively improve response relevance and accuracy. This approach was designed to address limitations observed in out-of-the-box LLMs, specifically their inability to reliably produce actionable local recommendations without supplementary domain context.” (Fig 2).

**Table 2:** Criteria for GPT-4 Prompt Design.

Prompt criteria	Detailed instructions
Clarity and Brevity	providing clear and concise responses, avoiding overwhelming users with excessive details, and adhering to brevity unless additional information is necessary
Data-Driven Responses	uses a structured database of autism care providers in Alabama, including service types, locations, and contact information, to accurately match user queries with service recommendations
Feedback Integration	After each interaction, Athena prompts users for feedback with a simple “Yes/No” question. This feedback is vital for continuous learning, refining responses based on user input to enhance effectiveness
Privacy/ Customization	respects user privacy, handling interactions without storing personal data unless explicitly authorized, and adheres to stringent privacy standards to ensure secure communications
Guided Interactions	asks direct, relevant questions for clarification, maintaining a supportive tone to precisely identify user needs without causing frustration

### Implementation of retrieval-augmented generation (RAG)

To further enhance the precision and transparency of the chatbot, we implemented a Retrieval-Augmented Generation (RAG) framework consisting of two main stages: document retrieval and response generation. For the retrieval stage, we employed the OpenAI text-embedding-ada-002 model to generate dense vector embeddings for provider profiles, capturing semantic relationships beyond keyword matching [65]. Each profile contained comprehensive details, including provider name, address, services offered, and contact information. These embeddings were stored in a vector database, enabling efficient similarity searches. When a user query was received, it was converted into an embedding, and cosine similarity scores were calculated between the query embedding and stored provider embeddings. Provider profiles with similarity scores exceeding a predefined threshold (0.5) were deemed relevant.

In the subsequent generation stage, relevant documents retrieved from the first stage were incorporated into prompts provided to the OpenAI GPT-3.5-turbo model. Generation parameters were explicitly set, including temperature (set to 0) and maximum tokens (set to 500), to optimize precision. The GPT-3.5-turbo assistant was explicitly instructed to generate outputs solely based on the retrieved information, enhancing transparency and minimizing hallucinations.

To further refine recommendations, geographical filtering was applied. User-provided locations were geocoded using the Nominatim geocoder from the geopy library. Provider locations were similarly geocoded, and the geodesic distance between user and provider locations was calculated. Providers located within a specified radius (50 km) were included in the results. Missing or incomplete geocoding data were handled by marking providers’ distances as “Unknown,” ensuring inclusivity in recommendations. Finally, a Flask-based web application provided a user-friendly interface, allowing users to input natural language queries and receive structured responses detailing provider names, distances, services offered, and contact information.

### Evaluation framework

The performance of the developed systems was evaluated from the perspective of social workers as the primary intended users, with the aim of assessing feasibility and comparative performance trends in providing accurate, contextually relevant, and actionable responses for autism-related service navigation queries. Evaluation focused on descriptive performance metrics, precision, recall, and F1 score, rather than formal statistical hypothesis testing, reflecting the proof-of-concept scope of this study. We developed a standardized set of test queries reflecting real-world scenarios encountered by families and social workers seeking autism services in Alabama. These queries were categorized into six criteria:

- Simple Queries: Single-service requests with minimal criteria.
- Complex Queries: Multi-criteria requests involving multiple services or conditions.
- Geographically Specific Queries: Requests targeting specific locations.
- Synonyms and Varied Phrasing: Queries using alternative terminology or colloquial expressions.
- Ambiguous Queries: Broad or unclear requests requiring interpretation.
- Less Common Services: Queries for specialized or rare services like equine therapy.

Each category included ten representative queries (e.g., “Find an Occupational Therapist in Huntsville, AL, who accepts Medicaid and works with young adults”). These queries were developed in collaboration with the Alabama Department of Mental Health to ensure relevance and practical alignment with user needs. The output query results were categorized as Correct Matches only if they fully satisfied all constraints in the query, including service type, geographic location, insurance acceptance, and age group where applicable. Partially correct matches (e.g., matching location and service but not insurance) were not counted as correct. These results were analyzed using precision (how many of the returned results were correct), recall (how many of the correct results were returned compared to the total possible correct results), and F1 score (the harmonic mean of precision and recall, balancing both metrics) [66]. These metrics were used to summarize system behavior across representative query types rather than to support inferential statistical comparisons. In the results section we report F1 scores directly, which summarize system performance across multiple query constraints.. This evaluation aimed to provide a descriptive, albeit semi-quantitative, performance comparison across the tested models (Traditional NLP, Out-of-the-box LLMs, Domain-specific LLM, and RAG-based system). Formal statistical significance tests (e.g., McNemar’s test, t-tests) were not conducted. Performance was evaluated using precision, recall, and F1 scores metrics across representative query types. Future evaluations will benefit from incorporating inferential statistical analyses to validate model performance more quantitatively. While we report precision, recall, and F1 scores as descriptive metrics of system performance, we did not conduct formal statistical significance testing (e.g., McNemar’s test, bootstrap confidence intervals) in this study. This choice reflects the proof-of-concept focus on feasibility rather than definitive benchmarking. Future work should incorporate such analyses to provide stronger inferential support for observed performance differences.


$$Precision=\frac{Correct\;Matches}{Total\;Matches\;Returned}\cdot Recall=\frac{Correct\;Matches}{Total\;Matches\;Available}\cdot F\text{1}\;Score=\text{2}\times \frac{Precision\times Recall}{Precision+Recall}$$

## Results

### Socioeconomic mapping of autism services

**Fig 3:**
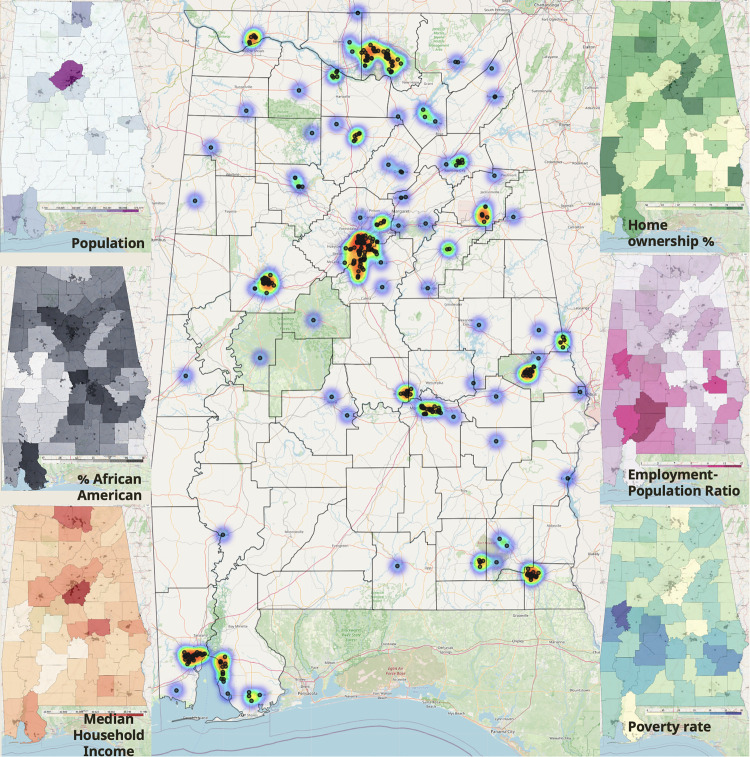
Geographic distribution of autism-related service providers in Alabama, overlaid on key socioeconomic indicators. Heatmap represents provider density. Insets show demographic context, including poverty rate, population distribution, and median income. The figure highlights service disparities that emerged during database construction and motivate the need for equitable access tools like Athena and Minerva. Base map data © OpenStreetMap contributors, available under the Open Database License (ODbL). Overlays and analysis by the authors.

The resulting spatial representation (Fig 3) of socioeconomic indicators allows for clear understanding of areas underserved by autism-related services. While this study’s primary aim was not to explore or explain these disparities, this analysis revealed disparities in the availability of autism-related services across Alabama. Regions with poverty rates exceeding 19% and minority populations above 20% demonstrated significantly fewer service providers per capita compared to the state average. Some counties lacked autism-specific providers altogether (Fig 3). These findings underscore inequities in resource distribution, suggesting a need for targeted interventions to address underserved areas. Although a range of factors may contribute to these service gaps [67], the maps suggest a potential need for a data-driven approach to improve resource allocation.

### Evaluation of the NLP-driven chatbot

The initial NLP-driven chatbot was evaluated to assess its capability to process autism-related service queries (Table 3), with results interpreted descriptively to highlight observed performance patterns rather than statistically significant differences. This system employed traditional NLP techniques, including syntactic processing and rule-based matching, consistent with approaches commonly used in existing service directories. Its performance therefore reflects realistic limitations of deployed keyword-based systems rather than deficiencies arising from artificial model simplification. Performance is reported using the F1 score, defined as the harmonic mean of precision and recall. These approaches required predefined tags and depended on exact matches or cosine similarity to link user queries to services.

**Table 3:** Performance Metrics of the NLP-Driven Chatbot.

Sample Query	Performance Score (F1)
Find an Occupational Therapist (OT) and Speech Therapist in Huntsville, AL who accepts Medicaid and works with 20-year-olds.	63.2%.
Locate a Speech Therapist in Birmingham, AL specializing in early intervention and accepting private insurance	74.5%.
Search for a Pediatric Neurologist in Montgomery, AL who offers telehealth services and accepts Medicare.	58.9%.

This method, while effective in certain scenarios, exhibited limitations in handling the nuances of natural language, particularly when users employed varied phrasings, idiomatic expressions and synonyms, or complex query intents which are reported in research articles [68] where the system struggled to accurately match these to the corresponding services. As a result in our case, the chatbot occasionally produced incomplete or less accurate responses, particularly in cases where the user’s language deviated from the predefined tags. The chatbot’s performance across previously randomly generated test queries of Table 3 shows where non-standard language was used, the F1 score of the responses dropped (Table 4).

**Table 4:** Challenges Faced by the NLP Chatbot in Query Processing.

Sample Query	Challenges	Performance Score (F1)
Therapist in Huntsville, AL who does OT and speech therapy for a young adult covered by Medicaid.	Uses “recommend a therapist” instead of directly specifying “Occupational Therapist (OT) and Speech Therapist,” and “young adult” instead of specifying age.	57.1%.
Where in Birmingham, AL can I find a specialist for early language intervention who works with private insurers?	Uses “specialist for early language intervention” instead of “speech therapist specializing in early intervention,” and “private insurers” instead of “private insurance	48.6%.
Are there pediatric neurologists in Montgomery, AL who offer virtual consultations and accept Medicare?	Uses “virtual consultations” instead of “telehealth services” and “pediatric neurologists” is rephrased slightly as “Are there pediatric neurologists” instead of “Search for a pediatric neurologist,”	53.7%.

Table 5 presents the different aspects of the NLP system, including its handling of simple, complex, and geographically specific queries, as well as its ability to process synonyms, varied phrasing, ambiguous language, and requests for less common services highlighting where the system performed well and where it struggled.

**Table 5:** Scenario-Based Performance Evaluation of the NLP Chatbot.

Criteria	Query	Expected Service	Matched Service	Reason for Mismatch
Simple, straightforward queries	“Find a Speech Therapist in Birmingham, AL who accepts Medicaid.”	Speech Therapist in Birmingham accepting Medicaid	Speech Therapist in Birmingham accepting Medicaid	Exact Match
Complex queries with multiple criteria	“I need an Occupational Therapist and a Speech Therapist in Huntsville, AL who accept Medicaid and specialize in working with young adults aged 20.”	OT and Speech Therapist in Huntsville accepting Medicaid	Only Speech Therapist in Huntsville accepting Medicaid	Couldn’t match both OT and Speech Therapist together
Queries with synonyms or varied phrasing	“Looking for a language specialist in Birmingham, AL who handles early intervention and takes private health insurance.”	Speech Therapist specializing in early intervention	General Speech Therapist	Synonym “language specialist” not correctly linked to “Speech Therapist”
Geographically specific queries	“Are there any pediatric neurologists near downtown Montgomery, AL who offer telehealth services and accept Medicare?”	Pediatric Neurologist in Montgomery offering telehealth	Pediatric Neurologist in Montgomery	Location specificity not considered effectively by NLP system
Ambiguous or incomplete queries	“Can I find someone in Alabama who helps with autism?”	Autism specialist or center	Various unrelated services	Ambiguous phrasing, system unable to accurately determine intent
Queries with less common or rare services	“Where in Mobile, AL can I find an equine therapy program for children with autism that accepts Medicaid?”	Equine therapy program for children in Mobile accepting Medicaid	General therapy services in Mobile	Rare service not sufficiently represented in training data

Table 6 shows the distribution of the results of each query type (averaged on ten queries), alongside calculated precision, recall and F1 score. The NLP chatbot performed well on simple, single-service requests with direct phrasing while queries requiring semantic understanding, multi-criteria matching, or complex phrasing resulted in lower performance suggesting that the traditional NLP methods are effective for straightforward tasks but lack the semantic depth and contextual awareness needed for nuanced or highly specific queries.

**Table 6:** Precision, Recall, and F1 Scores of the NLP Chatbot.

Criteria	Correct Matches	Partially Correct Matches	Incorrect Matches	Precision (%)	Recall (%)	F1 Score
Simple, straightforward queries	7	2	1	87.5	70.0	77.8
Complex queries with multiple criteria	5	3	2	71.4	50.0	58.8
Queries with synonyms or varied phrasing	4	3	3	57.1	40.0	47.0
Geographically specific queries	6	2	2	75.0	60.0	66.7
Ambiguous or incomplete queries	3	2	5	37.5	30.0	33.3
Queries with less common or rare services	2	1	7	22.2	20.0	21.0

### Performance of out-of-the-box LLMs

Generative AI models were evaluated using the same query set (Table 7). While these models exhibited superior language comprehension and handled nuanced queries better than the NLP chatbot, they struggled with regional specificity and consistency.

**Table 7:** Comparison of Established LLMs for Autism Resource Queries.

Sample Query	Performance Score (F1)			
	ChatGPT (%)	Google Gemini (%)	Microsoft Copilot (%)	Claude (%)
Find an Occupational Therapist (OT) and Speech Therapist in Huntsville, AL who accepts Medicaid and works with 20-year-olds.	47.8	51.6	42.3	0.0
Locate a Speech Therapist in Birmingham, AL specializing in early intervention and accepting private insurance	43.2	37.5	33.7	2.1
Search for a Pediatric Neurologist in Montgomery, AL who offers telehealth services and accepts Medicare.	57.9	46.3	48.6	3.4

**Fig 4:**
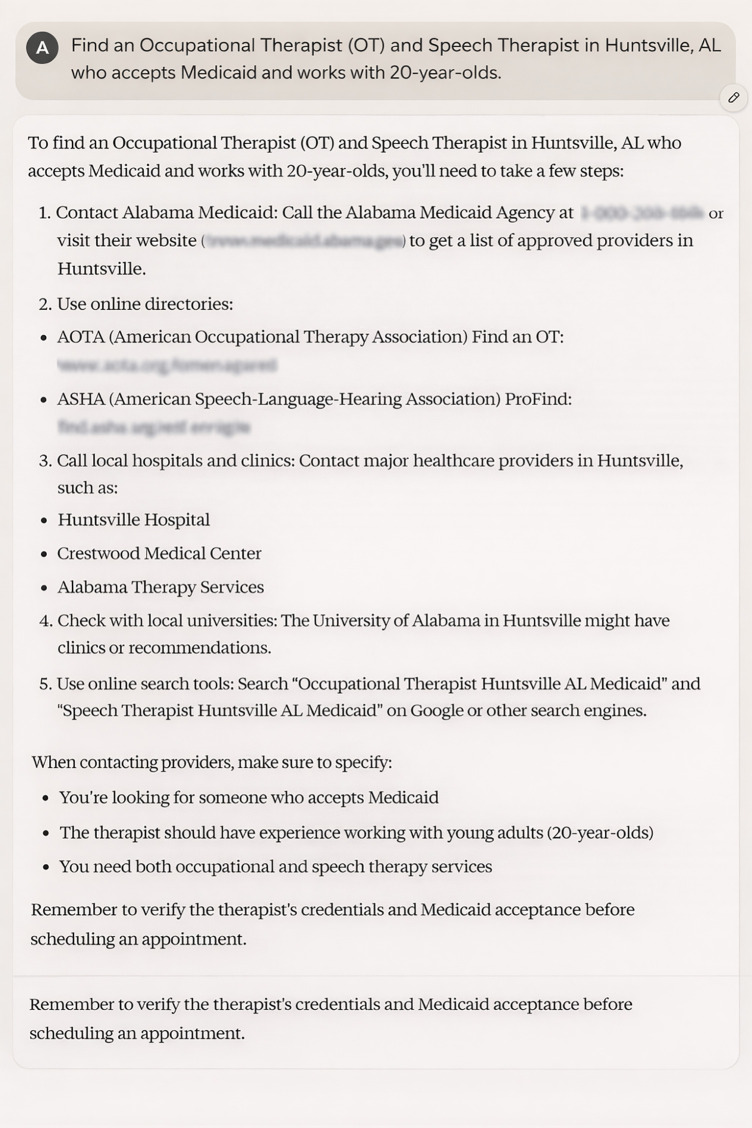
Example of LLM Fails to Provide Relevant Information.

For instance, they were able to parse queries involving multiple provider types or specific insurance requirements without difficulty. Their broad knowledge base and advanced linguistic capabilities allowed them to interpret complex sentence structures and varied phrasing more effectively than the rule-based approach previously used. But despite their language processing abilities, the out-of-the-box LLMs struggled to provide region-specific or highly specialized information related to autism care in Alabama. For example, they often failed to distinguish between service providers who were available locally and those who were not. As an example, ChatGPT’s failure to understand the local context led it to suggest service providers that were either not in Alabama or did not specialize in autism care, providing generic advice rather than specific, actionable information. Or Microsoft Copilot’s lack of awareness of regional service providers resulted in recommendations that were irrelevant to the user’s location, often suggesting national organizations or services unavailable in Alabama. In several cases, the models returned generic responses rather than the specialized advice expected in the autism care context. For instance, in answer to a query on finding an OT and speech therapist in Huntsville, AL, Claude gives general suggestions like contacting Alabama Medicaid, doing an online search, or contacting local universities for recommendations (Fig 4). Also, the outputs might not be consistent, if a query is being asked multiple times, each time a different answer might show up. For instance Table 8, shows a query that was asked (verbatim) five times from Gemini LLM and each time a different response was generated by LLM.

**Table 8:** Inconsistencies in LLM Responses to Identical Queries.

Query	Summary of Response
Find two Occupational Therapist (OT) and Speech Therapist in Huntsville, AL who accepts Medicaid and works with 20-year-olds.	1- General suggestions on finding therapists, including contacting Medicaid providers, local hospitals, universities, and online directories like Beaming Health.
	2- Provides names of specific therapy centers (Child’sPlay, Therapy Achievements, Pediatric Outpatient Center) that accept Medicaid and suggests further searches.
	3- Suggests using Beaming Health, contacting Medicaid providers for in-network therapists, and checking with local hospitals or rehab centers for therapists.
	4- Lists specific therapy centers (Child’sPlay, More Than Words, Pediatric Therapy Link) that accept Medicaid and work with 20-year-olds.
	5- Provides a list of therapy centers with addresses on Google Maps but recommends contacting them directly to confirm Medicaid acceptance for 20-year-olds.

Interestingly, in terms of region-specific accuracy, the domain-focused NLP chatbot (Athena) we developed using traditional NLP methods outperformed the LLMs. This indicates that while LLMs offer considerable potential for general tasks, their utility in highly specialized, context-heavy domains like autism care may be limited without additional context or training on domain-specific data. The LLMs often returned less accurate results when asked for specific services that required deep local knowledge, especially when compared to our earlier system, which was trained on curated, location-specific datasets.

### Domain-specific LLM-based chatbot testing

The customized GPT-4-based chatbot, Athena, demonstrated substantial improvements over both traditional NLP and out-of-the-box LLMs (Table 9, 10).

**Table 9:** Performance Comparison: NLP vs. LLMs.

Query	NLP System Result	LLM Result	Observation
“Find a provider for ABA therapy in Birmingham”	Partial match; misses some relevant providers	Comprehensive match with all relevant providers	NLP struggled with synonyms, LLM recognized intent
“Looking for speech therapy near Montgomery”	No relevant results	Accurate and relevant results	NLP failed to match non-exact phrasing, LLM succeeded
“Are there providers for OT in Huntsville?”	Accurate but limited	Accurate and more comprehensive	NLP provided limited results, LLM covered more options
“Who accepts Medicaid for ABA in Mobile?”	Incorrect match (focused on location only)	Correct match with relevant insurance filter	NLP failed to account for insurance filter, LLM did well

**Table 10:** Performance Metrics (F1 Score) Metrics for Different Models.

Sample Query	NLP Accuracy (%)	Out-of-the-Box LLM Accuracy (%)	Domain-Specific LLM Accuracy (%)
Find an Occupational Therapist and Speech Therapist in Huntsville, AL who accepts Medicaid.	66.7	44.9	91.2
Locate a Speech Therapist in Birmingham specializing in early intervention.	72.1	38.4	93.7
Search for a Pediatric Neurologist in Montgomery offering telehealth services.	61.3	56.5	89.1

More granular comparison of these models by comparing F1 scores across different scenarios also shows domain specific LLM-based chatbot outperforms the NLP based (Table 11).

**Table 11:** Scenario-Specific Performance Across NLP and Athena.

Criteria	Traditional NLP	Domain-Specific LLM
Simple Queries	79.4	94.6
Complex Queries	51.2	89.5
Queries with Varied Phrasing	42.7	86.3
Geographically Specific Queries	58.9	87.4
Queries for Less Common Services	23.5	81.7

Although the numbers reflect qualitative assessments rather than precise, scientifically measured values, these results indicate that while traditional NLP systems generally struggled with varied phrasing and complex queries, out-of-the-box LLMs showed improvement but still lacked the precision and recall necessary for highly specialized autism care services. In contrast, using domain-specific tuning of GPT-4 consistently outperformed both methods which highlights a clear improvement in relevance and response accuracy, even if the evaluations are based on observed trends rather than rigid statistical metrics.

### Performance of RAG-based chatbot

While the GPT-4-powered Athena chatbot demonstrated significant improvements in handling nuanced queries and domain-specific autism care services, it relied on a custom version of OpenAI’s ChatGPT with limited control over model parameters such as temperature, token limits, and conversation context [69]. To enhance functionality and precision, we implemented a RAG approach to address the shortcomings of traditional NLP and out-of-the-box LLMs by integrating large language models with a dynamic retrieval mechanism from our curated dataset of autism service providers [70]. This provides greater control over model parameters, enabling fine-tuning of responses to meet user expectations. By integrating real-time retrieval from our curated dataset, the chatbot provides contextually relevant and up-to-date information. Responses are based on specific retrieved documents, allowing us to trace the source of information, which is crucial in healthcare applications for building user trust. Comparative performance observation of these models (Table 12) shows RAG enhanced semantic matching, enabling precise recommendations even for complex, multi-criteria queries. In addition to these qualitative comparisons, RAG offers superior control over system performance metrics.

**Table 12:** Comparative Observations: RAG-Based vs. GPT-Based Athena.

Sample Query	Athena GPT-4 Result	RAG-Based Athena Result	Observation
Find an Occupational Therapist and Speech Therapist in Huntsville, AL who accepts Medicaid.	Partial match	Accurate and specific match	RAG retrieved more precise data on providers and services.
Locate a Speech Therapist in Birmingham specializing in early intervention.	General match	Specific match	RAG incorporated specific local data not available to GPT-4 alone.
Search for a Pediatric Neurologist in Montgomery offering telehealth services.	Incomplete match	Full match	RAG handled multiple criteria more effectively.

While GPT-4-based system performed well in complex queries, RAG’s structured retrieval mechanism resulted in even higher accuracy, precision, and recall (Table 13). For example, across complex multi-criteria queries, the RAG-based system achieved an F1 score of 94.3 compared to 85.2 for the GPT-4-based chatbot, reflecting a clear performance gain.

**Table 13:** Precision and Recall of RAG-Based vs. GPT-Based.

Criteria	GPT-4-Based Precision (%)	RAG-Based Precision (%)	GPT-4-Based Recall (%)	RAG-Based Recall (%)
Simple Queries	91.3	96.2	87.5	97.2
Complex Queries	87.1	94.3	83.3	95.5
Geographically Specific Queries	84.6	92.7	79.2	93.1

Across all query types, the RAG-based system consistently outperformed the GPT-4-based chatbot, with improvements of 5–8 percentage points in precision and 10–12 percentage points in recall. These gains were most pronounced for complex queries (RAG: 94.3% precision, 95.5% recall vs. GPT-4: 87.1% precision, 83.3% recall), underscoring the robustness of the RAG framework in handling multi-criteria and geographically specific requests. This quantitative margin substantiates our characterization of the RAG model as achieving superior performance over both traditional NLP systems and general-purpose LLMs. In comparison to the custom GPT-4-based system, which was reliant on predefined knowledge, this RAG-based architecture provides Enhanced Control, Improved Accuracy, Transparency, and Explainability. With RAG, we can adjust critical generation parameters such as temperature (for controlling the randomness of output), token limit (for managing the length of responses), and the number of conversation turns kept in context [71]. The ability to incorporate real-time retrieval from our own curated dataset means that the RAG-based model is not limited by the pre-trained knowledge of an LLM. It can dynamically pull the most relevant information from a vast repository of autism service data, ensuring that responses are not only contextually relevant but also accurate and up-to-date. With RAG, every response is based on retrieved documents, allowing us to trace the origin of the information. This transparency is a key differentiator when compared to Athena’s GPT-4-based responses, where the sources of information were more opaque. For sensitive healthcare domains like autism services, the ability to verify and explain responses is crucial for user trust.

## Conclusion and discussion

This study systematically explored the potential of AI-driven tools for improving autism-related healthcare resource management in Alabama. By developing and evaluating several chatbot models—including traditional NLP methods, out-of-the-box large language models (LLMs), a customized domain-specific GPT-4 chatbot, and a Retrieval-Augmented Generation (RAG) chatbot—we were able to clearly demonstrate the feasibility and comparative strengths of different AI approaches in this specialized social work context. All evaluations were conducted from the perspective of social workers as the primary users, reflecting the system’s intended role as a decision-support and navigation aid within professional care coordination workflows.

Among the systems evaluated, traditional NLP-based methods showed effectiveness for simple, clearly structured queries, achieving up to 87.5% precision and 70% recall. However, these models were limited by their reliance on exact keyword matching and struggled to handle complex, multi-criteria queries and linguistic variations. Out-of-the-box LLMs, such as ChatGPT, Google Gemini, Microsoft Copilot, and Claude, exhibited strong capabilities in understanding nuanced, natural language queries but faced significant limitations in terms of geographic specificity, consistently returning generic and less actionable recommendations due to the absence of localized, domain-specific training. In contrast, our domain-specific GPT-4 chatbot (Minerva) substantially improved upon these approaches by effectively leveraging structured local datasets, clearly highlighting the importance of domain-specific fine-tuning. This model achieved significantly higher F1 scores (approximately 85–95%) across diverse query types, demonstrating its utility in real-world scenarios faced by social workers and caregivers. Finally, the Retrieval-Augmented Generation (RAG) chatbot emerged as the strongest model, integrating real-time retrieval of relevant provider information with generative AI capabilities. It provided enhanced accuracy (approximately 90–96% precision and recall), improved transparency, and higher explainability of recommendations. These characteristics are particularly valuable for sensitive healthcare applications where trust and interpretability are critical. Importantly, the traditional NLP baseline was included to represent a practical reference point rather than a competitive benchmark, highlighting the performance gains achievable when transitioning from commonly used directory-style tools to more advanced LLM- and RAG-based systems.

A primary limitation of this study is its proof-of-concept design, which relied on descriptive performance metrics and exploratory comparisons rather than formal statistical significance testing. Additionally, the practical deployment of these AI tools faces inherent challenges, including the need for continuous updating of service provider information, potential computational costs associated with maintaining vector embeddings, and ethical considerations surrounding algorithmic biases and data privacy. Our results clearly underscore the promise and practicality of tailored AI tools—particularly domain-specific GPT-4 and RAG-based systems—for enhancing social work practices related to autism care. However, successful real-world implementation will require thoughtful integration strategies, regular data management practices, and attention to ethical and operational considerations. Observed marked disparities in the geographic and socioeconomic distribution of autism-related services across Alabama were observed (Fig 3). These disparities, while not the central focus of this study, illustrate the need for intelligent assistive tools that can help families and social workers identify appropriate resources despite systemic access gaps, when identified.

While the findings of this study demonstrate the potential of AI-driven tools in autism care, several important ethical and regulatory considerations must be addressed to ensure responsible deployment and real-world impact. One primary concern is algorithmic bias. AI models, particularly large language models (LLMs), risk reinforcing existing disparities if trained on data that inadequately represents marginalized populations. This is particularly relevant in autism services, where racial and socioeconomic disparities are well-documented [67]. LLMs have also shown susceptibility to inheriting biases present in their training data, potentially impacting equitable service recommendations [43]. Data privacy and security are equally critical, especially when handling sensitive healthcare information. Compliance with regulations like the Health Insurance Portability and Accountability Act (HIPAA) is essential to protect personally identifiable information. Although this study utilized mostly publicly and non-sensitive available service provider data, future implementations involving patient data must incorporate privacy-preserving techniques such as de-identification and encryption [72]. From a regulatory standpoint, ensuring model transparency, explainability, and human-in-the-loop review processes will be necessary for ethical compliance and user trust [73]. Moreover, continuous auditing and bias assessment should be embedded into system maintenance to mitigate risks of harm or unequal service delivery.

While as a pilot, this study demonstrates the potential framework for an AI-driven system to support autism care resource management, practical implementation would require further development to ensure adaptability to changes in available services and usability by intended stakeholders. Future iterations should incorporate mechanisms for periodic database updates to reflect changes in provider availability, service offerings, and insurance coverage. This could involve a combination of automated data collection, manual verification, and partnerships with state agencies to maintain accuracy over time [4]. The system is proposed to be designed to support accessibility through a web-based interface capable of processing natural language queries. Social workers, families, and caregivers represent primary user groups. Targeted training materials, including user guides and interactive onboarding, would be necessary to ensure effective engagement, particularly for non-technical users. Prior studies emphasize that such training is essential to maximize the utility of AI tools in social work and healthcare environments [26]. While this study emphasized natural language interfaces to assess the feasibility of LLM-powered semantic search and to be used by users with high digital literacy. However, traditional UI designs (e.g., dropdowns, filters) may offer complementary benefits in terms of simplicity or accessibility for some user groups. Future work could compare chatbot-driven interfaces with conventional UI approaches to assess their relative performance, user preferences, and usability across diverse populations. Finally, incorporating user feedback mechanisms can enable continuous refinement of recommendations and system performance. Feedback loops are increasingly recognized as a best practice in AI system design to enhance accuracy, relevance, and user trust [74].

Together, these considerations emphasize that while AI-driven tools hold significant promise for improving autism care resource navigation, their real-world deployment will require careful attention to ethical, regulatory, and practical implementation factors. This proof-of-concept study provides a foundation for future research and development aimed at operationalizing such tools within social work and healthcare systems.

## Supporting information

S1 Table Stakeholder Roles and Service Mapping. (DOCX)
