# Supplementary material for: Using AI to enhance healthcare resource management and allocation: A focus on the autism community in Alabama
Source: PLoS One. 2026 Mar 16;21(3):e0342700. doi: 10.1371/journal.pone.0342700 (PMC12991235; doi:10.1371/journal.pone.0342700)
Supplement: S1 Table — (DOCX) [file pone.0342700.s001.docx]

**Supplementary data:**

**Table S1**: Stakeholder Roles and Service Mapping

| **Organization Type** | **Primary Function** | **Primary Provider** | **Secondary Provider(s)** | **Primary Beneficiary** |
| --- | --- | --- | --- | --- |
| Art Therapist | Art-based skill development | Art therapist | Family, Various therapists | Child with autism |
| Autism Advocacy Organizations | Advocacy and policy making | Advocacy workers | Volunteers, Lawyers | Autism community |
| Autism Research Centers | Research and development | Researchers | Technicians, Volunteers | Autism community |
| Autism Support Group | Peer support and resources | Group coordinator | Families, Therapists | Child with autism and family |
| Behavioral Therapist | Behavioral assessment and intervention | Behavioral therapist | Family, Various therapists | Child with autism |
| Clinical Psychologist | Assessment and treatment | Clinical psychologist | Family, Therapists | Child with autism |
| Early Intervention Services | Early childhood intervention | Early intervention specialists | Family, Various therapists | Child with autism |
| Family | Support and care | Parents or siblings | None | Child with autism |
| Government Health Agencies | Policy making and funding | Government officials | Various health professionals | Autism community |
| Government Health Department | Health policy making and implementation | Government health officials | Various health professionals | Autism community |
| Insurance Companies | Coverage of treatments and services | Insurance agents | None | Family with autistic child |
| Insurance Companies | Insurance policy making and implementation | Insurance policy makers | None | Family with autistic child |
| Legal Services | Legal support and advice | Lawyers | None | Family with autistic child |
| Local Community Centers | Community support and resources | Community center staff | Volunteers, Various therapists | Autism community |
| Local Government | Local policy making and implementation | Local government officials | Local health and education professionals | Autism community |
| Music Therapist | Music-based skill development | Music therapist | Family, Various therapists | Child with autism |
| National Government | National policy making and implementation | National government officials | Various health and education professionals | Autism community |
| Neurologist | Medical assessment and treatment | Neurologist | Family, Therapists | Child with autism |
| Nutritionists | Diet and nutrition advice | Nutritionist | None | Child with autism |
| Occupational Therapist | Skill development | Occupational therapist | Family, Clinical psychologist | Child with autism |
| Pediatrician | General health care | Pediatrician | Family, Various therapists | Child with autism |
| Pharmacies | Medication supply | Pharmacists | None | Child with autism |
| Physical Therapist | Physical skill development | Physical therapist | Family, Various therapists | Child with autism |
| Public Health Departments | Public health services and policy making | Public health officials | Various health professionals | Autism community |
| Residential Service Providers | Residential care and support | Residential staff | Support staff, Therapists | Adult with autism |
| School Administration | School policy making and implementation | School administrators | Teachers, Therapists | Child with autism |
| Social Worker | Case management and support | Social worker | Various therapists, Family | Child with autism and family |
| Special Education School | Education and support | Special education teachers | Teaching assistants, Therapists | Child with autism |
| Speech Therapist | Language and communication skills development | Speech therapist | Family, Clinical psychologist, Occupational therapist | Child with autism |
| Technology Solution Providers | Technological aids provision | Tech developers | Support staff | Child with autism |
| Vocational Training Providers | Vocational skills training | Vocational trainers | Support staff | Adult with autism |
